# Potential Biomarkers for NSAID-Exacerbated Respiratory Disease

**DOI:** 10.1155/2017/8160148

**Published:** 2017-08-09

**Authors:** Hanki Park, Youngwoo Choi, Chang-Gyu Jung, Hae-Sim Park

**Affiliations:** ^1^Department of Allergy and Clinical Immunology, Kyungpook National University School of Medicine, Daegu, Republic of Korea; ^2^Department of Allergy and Clinical Immunology, Ajou University Medical Center, Suwon, Republic of Korea

## Abstract

Asthma is a common chronic disease with several variant phenotypes and endotypes. NSAID-exacerbated respiratory disease (NERD) is one such endotype characterized by asthma, chronic rhinosinusitis (CRS) with nasal polyps, and hypersensitivity to aspirin/cyclooxygenase-1 inhibitors. NERD is more associated with severe asthma than other asthma phenotypes. Regarding diagnosis, aspirin challenge tests via the oral or bronchial route are a standard diagnostic method; reliable *in vitro* diagnostic tests are not available. Recent studies have reported various biomarkers of phenotype, diagnosis, and prognosis. In this review, we summarized the known potential biomarkers of NERD that are distinct from those of aspirin-tolerant asthma. We also provided an overview of the different NERD subgroups.

## 1. Introduction

NSAID-exacerbated respiratory disease (NERD) is characterized by adult-onset chronic rhinosinusitis (CRS) with nasal polyps, intense eosinophilic infiltration in the upper and lower airway mucosa, and severe symptoms of exacerbation in response to aspirin/cyclooxygenase- (COX-) 1 inhibitors [1]. A previous systematic review had reported a NERD prevalence of 7% among typical adult asthmatic patients and twice among patients with severe asthma [2]. NERD is therefore considered a risk factor for severe asthma [3, 4]. Among patients with CRS and nasal polyps, the prevalence of NERD was 8.7% and 9.7%, respectively [2]. NERD is associated with severe CRS with nasal polyps, recurrence after sinus surgery, and airway remodeling [5–7], suggesting that NERD causes severe asthma with CRS/nasal polyps.

NERD has a unique pathophysiology, with increased levels of lipid mediators, activated eosinophils, and mast cells, even without COX-1 inhibitor treatment. Thus, in most studies defining asthma endotypes, NERD has been identified as an independent endotype [8, 9]. However, all patients with NERD are not accompanied by severe asthma, and their clinical course is also known to be variable [10]. Confirmative diagnosis of NERD is based on provocation tests with aspirin. Oral aspirin challenge is considered the gold standard diagnostic method; however, its use is often limited by the risk of severe reactions during the test. The bronchial aspirin challenge is safer and consumes less time; however, it is limited by its low sensitivity [11]. In addition, oral or bronchial aspirin challenge test has limitations that cannot be used to predict the treatment or prognosis of NERD. Therefore, *in vitro* tests should be developed for diagnosing and monitoring NERD.

In this review, we summarized three groups of known noninvasive biomarkers that can distinguish NERD from aspirin-tolerant asthma (ATA): lipid mediators, inflammatory cells and cytokines, and genetic markers. In addition, we reviewed the subtypes of NERD and the related biomarkers for developing precision medicine in the future.

## 2. NERD as an Endotype of Asthma

Recently, many studies were conducted to distinguish asthma phenotypes and endotypes that affect diagnosis, treatment choice, and prognosis. A phenotype refers to “clinically observable characteristics,” and it is distinguished by clinical features, pathophysiological factors, response to treatment, prognosis, and so on [12]. An endotype is a subtype of a disease that is functionally and pathologically defined by a molecular mechanism or a treatment response [13]. Although there is no widely accepted method for endotyping, most studies have classified NERD as an endotype of asthma [9, 12]. NERD is known to be a late-onset asthma, as the first symptoms usually start at the age of 20 ~ 40 years; females are more affected, and it is not influenced by family history or geographic region [14]. Rhinitis is usually the first observed symptom followed by asthma, sensitivity to aspirin, and nasal polyps [15]. Patients with NERD presented with moderate to severe asthma (with frequent exacerbation) have poor lung function and require more frequent intubation and systemic steroid bursts [6].

The pathophysiological features of NERD include lipid mediator imbalance and intense eosinophilic inflammation. Proinflammatory cysteinyl leukotrienes (cysLTs) and prostaglandin (PG) D2 (PGD2) are known to be markedly upregulated in NERD, whereas PGE2 has been found to be constitutively decreased [16–18]. Patients with NERD have a higher number of mast cells and eosinophils infiltrating the upper and lower respiratory mucosa, even without exposure to COX-1 inhibitors and changes in tissue eicosanoid metabolism [19–21]. In NERD, cytokines and chemokines show a trend of Th2 immune response [22, 23].

## 3. Biomarkers of NERD

### 3.1. Lipid Mediators

The most reproducible and informative biomarker to distinguish NERD from ATA is a high-level urinary LTE4 (Table 1). LTE4 is the substance last metabolized in cysLTs. LTC4 and LTD4 are easily metabolized in the following stages, while LTE4 is released into the urine in a stable manner; it is therefore suitable for use as a biomarker [24]. The LTE4 levels in induced sputum and saliva are higher in NERD than in ATA [25–27]. However, urinary LTE4, which indirectly reflects the activity of cysLTs in the lungs, has been used to distinguish NERD from ATA in many studies [25, 28–36]. In addition, the nature of the urine specimen makes it easier to standardize the level of LTE4, and it has the advantage of noninvasiveness. The value of urinary LTE4 is increased, in the baseline as well as under aspirin or COX-1 inhibitor provocation, in NERD compared to ATA. Thus, baseline urinary LTE4 can be used as a biomarker to distinguish NERD from ATA. This phenomenon is present in both random urine and 24 h urine; recent studies on 24 h urine have reported an area under the curve (AUC) of 0.87 [35]. In addition, it was confirmed that the metabolites of urinary LTE4 were significantly different in NERD and ATA, even in studies that used metabolomics [37]. Urinary LTE4 can be used not only to distinguish between NERD and ATA but also to indicate the prognosis and treatment response. Urinary LTE4 is associated with a decrease in FEV1 during aspirin challenge in patients with NERD [38]. It has been reported that urinary LTE4 is significantly higher in patients with NERD who failed in aspirin desensitization than in patients who achieved aspirin desensitization successfully [36]. Although urinary LTE4 is also increased in allergic asthma, eosinophilic asthma, and severe asthma without NERD, it can be used as a biomarker in patients with NERD, as it shows a remarkable increase in NERD, compared to ATA; it can therefore be used for predicting treatment response and prognosis.

PGD2 and PGE2, which are counteracted by the products of cyclooxygenase, are known to be closely related to the pathogenesis of NERD, but their use as biomarkers is still limited. PGD2 is mainly secreted from mast cells and eosinophils and is known to act as a proinflammatory and bronchoconstrictive mediator through CRTH2 [39]. Baseline PGD2 has been observed to be significantly increased in induced sputum [26]. PGD2 metabolites in urine and blood are also increased after aspirin provocation [40–42]. However, these results differ among studies, and the range of overlap is wide; therefore, the use of PGD2 as a biomarker of NERD to distinguish it from ATA is limited. A previous study had reported that urinary PGD2 metabolites reflect the difference between tolerant and intolerant groups during aspirin desensitization in patients with NERD [36]. Further studies are required to validate the use of PGD2 as a biomarker for predicting the treatment response and prognosis of NERD. PGE2 is considered a key mediator in the pathogenesis of NERD. Unlike cysLTs or PGD2, it is known to have anti-inflammatory and bronchoprotective effects in airway inflammation. Inhaled PGE2 prevents bronchoconstriction and cysLT production in NERD [43]. Most urinary PGE2 metabolites are derived from COX-2, and several studies have demonstrated that airway tissues in patients with NERD showed impaired expression of COX-2 [44, 45]. Apart from one study that suggested decreased baseline PGE2 levels in NERD [33], most studies showed no significant differences in the levels of PGE2 or its metabolites between NERD and ATA groups, indicating that further investigations are needed to evaluate its use as a potential therapeutic target.

The lipid mediators, not the arachidonic acid metabolites, have also been studied as biomarkers of NERD, especially sphingolipid metabolites. Sphingolipid metabolites mediate cell growth, cell differentiation, cell death, and autophagy, and the dysregulation of sphingolipid metabolism could induce airway inflammation and bronchial hyperreactivity [46, 47]. Baseline levels of serum sphingosine-1-phosphate (S1P) and urine sphingosine were significantly increased in patients with NERD, and a significant correlation with a decrease in FEV1 has been observed after aspirin challenge [48]. Sphingolipid metabolites may be possible biomarkers for NERD, although further studies are needed to validate their use.

### 3.2. Inflammatory Cells and Cytokines

The cellular pathogenic mechanism in NERD involves an intense eosinophilic inflammation, in which Th2 immunity orchestrates the phenotype of eosinophilic asthma (Table 2). Based on these findings, various studies have reported the eosinophil count, eosinophil-related mediators, and Th2 cytokines as biomarkers of NERD. Sputum and blood eosinophil counts are biomarkers for asthma phenotype of airway eosinophilic inflammation [49]. NERD is characterized by phenotypes represented by adult-onset eosinophilic asthma. Furthermore, local eosinophilia has been observed in the nasal polyp tissues or the bronchial lavage fluid of patients with NERD as well as blood eosinophilia [21, 50–52]. Therefore, sputum and blood eosinophil counts are difficult to use as direct diagnostic biomarkers of NERD, but they are important as biomarkers in distinguishing eosinophilic inflammation, one of the pathogenesis of NERD. Sputum and blood eosinophil counts are biomarkers that are also useful in predicting asthma severity and response to therapy [53, 54]. Therefore, sputum and blood eosinophil counts are biomarkers that can be used to evaluate the severity of NERD and the response to therapy. Among all the mediators of Th2 immunity, the biomarker most associated with NERD is periostin. Periostin is an extracellular matrix protein that is known to regulate inflammation/remodeling of the asthmatic airway [55]. Periostin is known to be a surrogate marker of Th2 immunity [56]. In a study on 277 adult asthmatic patients, we showed that serum periostin was a useful biomarker of NERD and that it could be used as an index of blood/sputum eosinophilia and asthma severity [57]. This study showed that it is useful as a biomarker to predict NERD (*p* = 0.006) even after multivariate regression analysis, which is more efficient than predictions of severe asthma phenotype (*p* = 0.04). This suggests that periostin is a potential independent biomarker of NERD diagnosis. Various other Th2 cytokines and chemokines, including IL-4, IL-5, IL-13, IL-33, TSLP, GM-CSF, and eotaxin have been studied, and some studies have shown statistically significant differences in their levels [58, 59]. In addition, cytokines such as IL-6, IL-8, and IFN-r have also been associated with AERD [60, 61], although further studies will be needed to validate their clinical significances.

Platelet activation is associated with leukocytes, which promote the secretion of proinflammatory lipid mediators such as cysLTs in NERD. Platelet activation induces the expression of cell adhesion molecules on the extracellular surface, which bind to the leukocytes through P-selectin (CD62P)–P-selectin glycoprotein ligand 1, GPIIb/IIIa-Mac-1, and CD40 ligand (CD40L)–CD40 [62, 63]. Recent studies have reported an increased percentage of platelet-adherent leukocytes and platelet activation markers such as sP-selectin and sCD40L in the blood of patients with NERD. These phenomena contribute to the overproduction of cysLTs [64, 65]. Thus, activated platelet surface markers are possible biomarkers for NERD, although further studies are needed to validate their use.

### 3.3. Genetic Markers

Various genetic polymorphisms have been reported through genetic association studies of targeted genes (lipid mediators and inflammatory responses) associated with the pathogenesis of NERD. In addition, a number of genome-wide association studies (GWAS) and epigenetic studies have reported potential genetic markers that distinguish NERD from ATA (Table 3).

First, the prevalence of HLA-DPB1^∗^0301 was significantly higher in patients with NERD in a Polish population; the same results were obtained in a study on a Korean population as well [66–68]. The patients carrying this marker had higher prevalence of CRS/nasal polyps than those who had no marker. Furthermore, GWASs demonstrated significant association of two SNPs of HLA-DPB1 (rs1042151 and rs3128965) and susceptibility to NERD [69, 70], suggesting HLA-DPB1 may be a strong genetic marker for predicting the NERD phenotype.

Genetic polymorphisms related to arachidonic acid metabolism and their receptor have been reported in candidate gene association studies. Leukotriene C4 synthase (LTC4S) is an important enzyme involved in the production of cysLTs. The gene that encodes LTC4S has been extensively studied for variations; however, it has been found to vary widely, depending on ethnic groups. In the study in the Polish population, which was the first study, it was possible to distinguish between NERD and ATA [71]. However, a recent meta-analysis did not show any significant results in NERD; the only significant results were obtained in the ATA and Caucasian subgroups [72]. In a Korean study, ALOX5 (5-LO enzyme gene) ht1 [G-C-G-A] was found to be significantly higher in NERD than in ATA in the 4SNP (−1708G>A, 21C>T, 270G>A, and 1728G>A) [73]. Two groups of receptors, cysLT receptors (CysLTR1, CysLTR2) and PGE2 (EP1, EP2, EP3, and EP4) receptor polymorphisms, have been demonstrated differences in polymorphism between NERD and ATA [74–78].

Several genetic markers associated with eosinophil activation have been reported. CRTH2 in response to PGD2, and CCR3 in response to eotaxin and RANTES, could induce eosinophil activation and recruitment. Polymorphisms in CRTH2 (−466T>C) and CCR3 (−520T>C) were associated with NERD [79, 80] and higher levels of eotaxin 2, indicating that the two SNPs of CRTH2 and CCR may be potential genetic markers that represent eosinophil activation in the upper and lower airway inflammation in NERD. Mast cells were also found with genetic polymorphisms that distinguish between NERD and ATA. The genotype frequency of *FCER1G*-237A>G was significantly different in patients with NERD, and patients with ATA and *FCERIA*-344C>T and Fc*ɛ*R1*β*−109T>C polymorphisms were associated with staphylococcal enterotoxin-specific IgE antibodies [81, 82].

So far, most genetic markers have been reported based on pathogenesis only. No genetic markers have been identified repeatedly in different patient groups, except HLA-DPB1. Further studies on diverse populations are required.

## 4. NERD Subtypes and Their Biomarkers

Although NERD is an endotype of asthma, it has been found in diverse phenotypes. Not all patients with NERD exhibit CRS and nasal polyps. The severity and response to treatment varies among patients. Recently, two studies of clustering in NERD cohorts were reported. The first study clustered 201 patients with NERD using a latent class analysis including clinical data from questionnaires, spirometry, atopy traits, blood eosinophilia, and urinary LTE4 concentrations as observable variables and found 4 classes [51]: class 1 patients showed moderate asthma course; class 2 showed mild asthma course; and class 3 and 4 patients showed severe asthma course. Blood eosinophilia and high urinary LTE4 were shown to be biomarkers that helped in class differentiation, especially for class 1. We performed a two-step cluster analysis using 3 clinical criteria: atopy, CRS, and urticaria to identify phenotypic clusters. We found 4 subtypes: subtype 1 (NERD with CRS/atopy and no urticaria), subtype 2 (NERD with CRS and no urticaria/atopy), subtype 3 (NERD without CRS/urticaria), and subtype 4 (NERD with urticaria). Subtypes 1 and 2 showed more severe clinical courses with higher blood/sputum eosinophilia and frequent asthma exacerbation requiring systemic steroid burst [83]. Higher levels of urinary LTE4 were observed in subtypes 1 and 3. These findings suggested that the level of LTE4 was not only a strong biomarker of NERD; it could also be applied to specific subtypes and endotypes of NERD. Classifying NERD into subtypes using biomarkers such as urinary LTE4 will help in better management of NERD.

## 5. Conclusion

The most useful biomarker of NERD is urinary LTE4. Urinary LTE4 level can be used for distinguishing the phenotype (including subtypes) and for predicting the response to desensitization and prognosis. Sputum/blood eosinophil counts are also biomarkers that can be used to identify the endotype of NERD and to monitor the course of treatment. Serum periostin levels and HLA-DPB1 (^∗^0301 and genetic polymorphisms) are suggested as useful biomarkers for predicting NERD phenotypes.

## Figures and Tables

**Table 1:** Lipid mediators.

	Mediators and parameters	Biologic sample	Detection method	Baseline		Response after ASA provocation	
				Compared to ATA	Reference	Compared to ATA	Reference
	LTE4	Urine	Immunoassay	↑	[25, 28–34]	↑	[25, 28, 30, 31, 34]
			Mass spectrometry	↑	[35, 36]	↑	[36]
		Saliva	Immunoassay	↑	[25]	↑	[27]
		Induced sputum	Immunoassay	↑	[25]		
			Mass spectrometry	↑	[26]	↑	[26]
		Blood and urine	Untargeted metabolomic analysis	↑	[37]		
							
COX pathway	PGD2	Induced sputum	Immunoassay	↑	[32]		
			Mass spectrometry	↑	[26]		
	PGD2 metabolite	Spot urine	Immunoassay			↑	[31, 33]
			Mass spectrometry	↑	[41]	↑	[41]
		Blood	Mass spectrometry	↑	[40]	↑	[40, 42]
	PGE2	Spot urine	Immunoassay	↓	[33]		
							
Others	Sphingolipid metabolite	Blood	Mass spectrometry	↑	[48]	↓	[48]
		Spot urine	Mass spectrometry	↑	[48]		

ATA: aspirin-tolerant asthma; LO: lipoxygenase; LT: leukotriene; COX: cyclooxygenase; PG: prostaglandin.

**Table 2:** Cellular and cytokine markers.

	Mediators and parameters	Biologic sample	Detection method	Baseline		Response after aspirin provocation	
				Compared to ATA	Reference	Compared to ATA	Reference
Cell	Eosinophil	NALF	Morphological count of stained slide			↑	[52]
	Platelet-adherent leukocyte	blood	Flow cytometry	↑	[65]		
	Soluble platelet surface marker	blood	Immunoassay	↑	[64]		

Others	ECP	NALF	Immunoassay			↑	[27]
	Periostin	Blood	Immunoassay	↑	[57]		

ATA: aspirin-tolerant asthma; NALF: nasal lavage fluid; ECP: eosinophil cationic protein.

**Table 3:** Potential genetic markers.

	Gene	Polymorphisms	Patients	Ethnic group	Mechanism	Reference
Arachidonic acid metabolism	CYSLTR1	_634 C>T, _475 A>C, _336 A>G	NERD: 105, ATA: 110, NC: 125	Korean	CysLTR1 expression	[74]
	CYSLTR2	_819T > G, 2078C > T, 2534A > G	NERD: 134, ATA: 66, NC: 152	Korean	CysLTR2 expression, LTC4S gene interaction	[75]
	EP2	uS5, uS5b, uS7	NERD: 198, ATA: 282, NC: 274	Japanese	Decrease transcription level of EP2, PGE2 braking	[76]
	PTGER	PTGER2: _616 C>G, _166 G>A PTGER3: _1709 T>A, PTGER4: _1254 A>G	NERD: 108, ATA: 93, NC: 140	Korean	PGE2, TXA2 receptor polymorphism	[77]
	TBXA2R	−4684C>, 795T>C				
	PTGER	PTGER3: rs7543182, rs959	NERD: 243, ATA: 918	Korean	PGE2 receptor polymorphism	[78]

Eosinophil-associated gene	CRTH2	_446T > C	NERD: 107, ATA: 115, NC: 133	Korean	Decrease CRTH2 expression and increase eotaxin-2 production	[79]
	CCR3	_520T > C	NERD: 94, ATA: 152	Korean	Higher mRNA expression of CCR3	[80]

HLA	HLA-DPB1	DPB1^∗^0301	59 NERD, 57 ATA, 48 NC	Polish		[66]
	HLA-DPB1	DPB1^∗^0301	76 NERD, 73 ATA, 91 NC	Korean		[68]
	HLA-DPB1	rs3128965	264 NERD, 387 ATA, 238 NC	Korean		[70]
	HLA-DPB1	rs1042151	117 NERD, 685 ATA	Korean		[69]

NERD: NSAID-exacerbated respiratory disease; ATA: aspirin-tolerant asthma; CysLTR: cysteinyl leukotriene receptor, LT: leukotriene; PG: prostaglandin; TX: thromboxane; CRTH: chemoattractant receptor homologue expressed by type 2 helper T cells; CCR: chemokine receptor; HLA: human leukocyte antigen; DPP: dipeptidyl peptidase.
